# Delayed Diagnosis of Osteonecrosis of the Femoral Head After a Minor Running Injury in a Healthy 28-Year-Old Man: A Case Report

**DOI:** 10.7759/cureus.115773

**Published:** 2026-09-04

**Authors:** Taehee Yoon

**Affiliations:** 1 Department of Rehabilitation Medicine, Haneul Hospital, Seoul, KOR

**Keywords:** adolescent and young adult, avascular osteonecrosis, delayed diagnosis, magnetic resonance imaging (mri), osteonecrosis of the femoral head

## Abstract

Osteonecrosis (avascular necrosis, AVN) of the femoral head in young adults is typically associated with high-energy trauma, corticosteroids, or alcohol abuse. Early disease is typically radiographically occult. We report a case of a previously healthy 28-year-old man who developed persistent right hip pain following a minimal twisting trauma while running. Initially obtained hip radiographs at an outside facility immediately following the trauma were interpreted as normal; the pain was considered to represent a soft tissue injury, and the patient underwent only a brief course of physical therapy and did not follow up further with the normal radiographs. However, the pain continued for seven months and was still preventing him from running. Therefore, the patient presented to our department, and a pelvic radiograph demonstrated mild osteoarthritic change of the right hip joint and features suspicious for osteonecrosis of the right femoral head. Further imaging with MRI confirmed osteonecrosis of the right femoral head with bone marrow edema, representing Ficat-Arlet stage II disease, with mild osteoarthritic change, chondromalacia, synovitis, and a large joint effusion in the right hip joint; no musculotendinous abnormalities were identified. The patient was referred to an orthopedic surgeon who recommended operative treatment. This case demonstrates that osteonecrosis of the femoral head can present in young healthy adults in the setting of seemingly trivial trauma, and normal early radiographs do not rule out the diagnosis. As a single case report, this does not establish causality.

## Introduction

Osteonecrosis of the femoral head is a progressive condition characterized by ischemia and death of subchondral bone due to impairment of the blood supply to the femoral head, resulting in collapse of the articular surface and secondary osteoarthritis [1,2]. It is especially common in young and middle-aged adults, where joint-preserving treatment should be favored over total hip arthroplasty [2,3]. Established risk factors of osteonecrosis include corticosteroids, excessive alcohol intake, and trauma [1,4]. However, traumatic osteonecrosis is commonly associated with high-energy trauma, such as femoral neck fractures and hip dislocations, with mechanical injury to the vessels supplying the femoral head [5].

A significant clinical challenge of osteonecrosis is its insensitivity to radiography in early stages. According to the Ficat-Arlet classification, stage I disease is radiologically normal, and stage II changes can be subtle [6,7]; early stages are also defined in several other popular classification systems [8,9]. MRI is very sensitive for detecting osteonecrosis with changes in bone marrow and is used for early diagnosis [10-12]. Normal radiographic examination at the early stage may be a reason for attributing pain to soft-tissue injury, with osteonecrosis being underdiagnosed until it reaches the advanced stage.

We present a young man in whom osteonecrosis of the femoral head was diagnosed seven months after a minimal running injury when normal initial radiographs suggested a soft-tissue injury as the cause of the problem. The temporal sequence described here does not establish causality.

## Case presentation

A 28-year-old man presented to our rehabilitation medicine department with right hip pain of seven months' duration. The pain started after a minimal twisting injury to the hip during running. He did not have any history of corticosteroid use, excessive or chronic alcohol abuse, smoking, systemic diseases, hip pathology, or a family history of osteonecrosis, and had normal laboratory findings.

Shortly after the injury, the patient was examined at another clinic, where his initial hip radiographs were interpreted as normal. Due to the minimal energy trauma, the pain was considered to be a consequence of soft tissue injury, and a short course of physical therapy was prescribed. Reassured by the normal radiographs, the patient decided not to continue his medical treatment and monitored his condition at home. Nevertheless, the pain continued to bother him for about seven months and interfered with running but was not limiting the performance of the activities of daily living.

**Figure 1:**
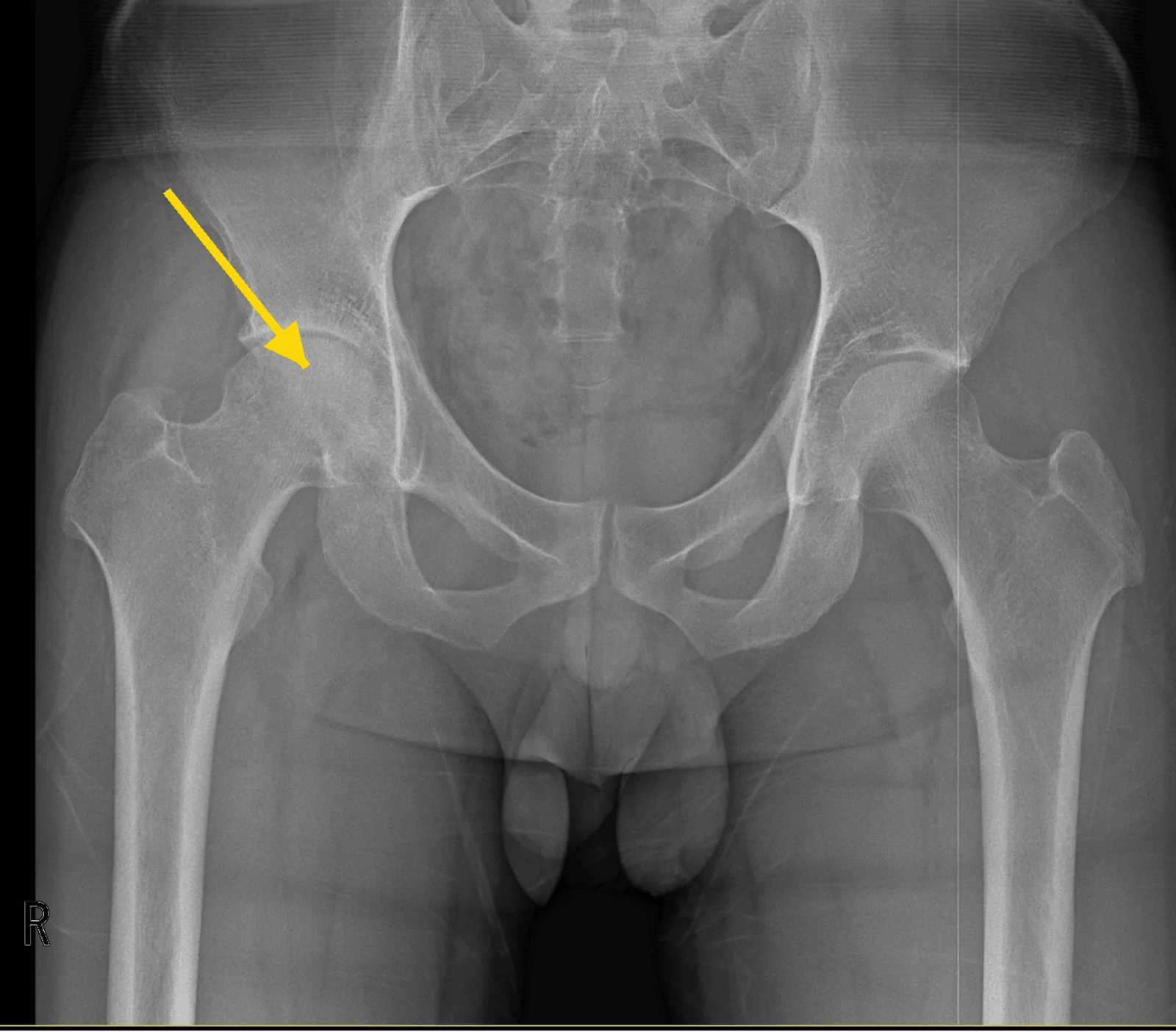
Anteroposterior pelvic radiograph at presentation The arrow indicates the superior articular surface of the right femoral head, showing mild osteoarthritic change and features suspicious for osteonecrosis.

On presentation to our department, the patient had a limited right hip range of motion: flexion (90°), external rotation (20°), extension (20°), and internal rotation (35°). On the contrary, he had a full range of motion in the left hip. Like the pain, these restrictions did not interfere with activities of daily living. The anteroposterior pelvic radiograph (Figure 1) demonstrated mild osteoarthritic change of the right hip joint and suspicious features for osteonecrosis of the right femoral head, compared to the normal initial radiographs taken seven months ago. There were no signs of subchondral collapse or crescent sign.

**Figure 2:**
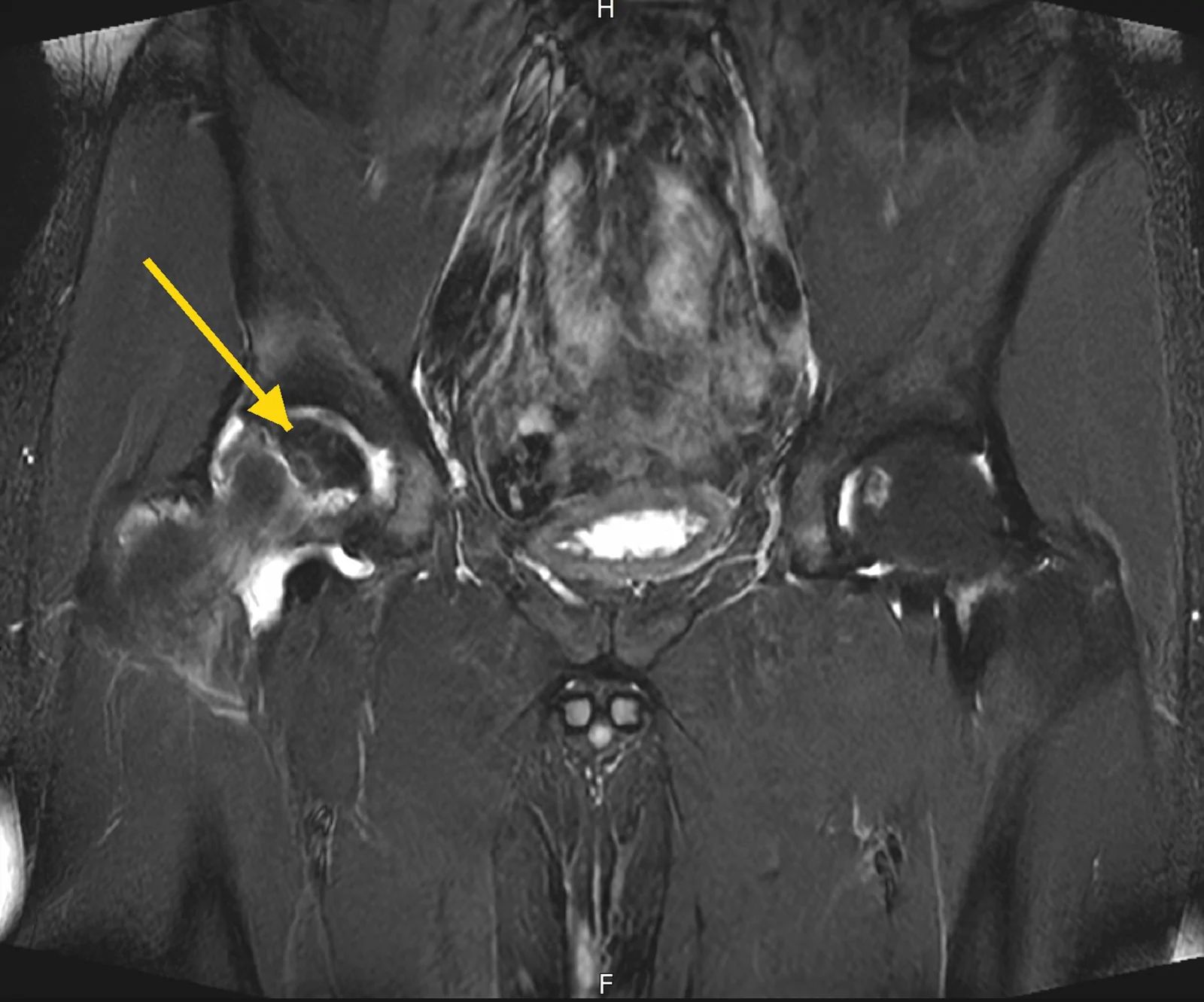
Coronal fat-suppressed MR image The arrow indicates the right femoral head with bone marrow edema; a large joint effusion and synovitis are present in the right hip.

**Figure 3:**
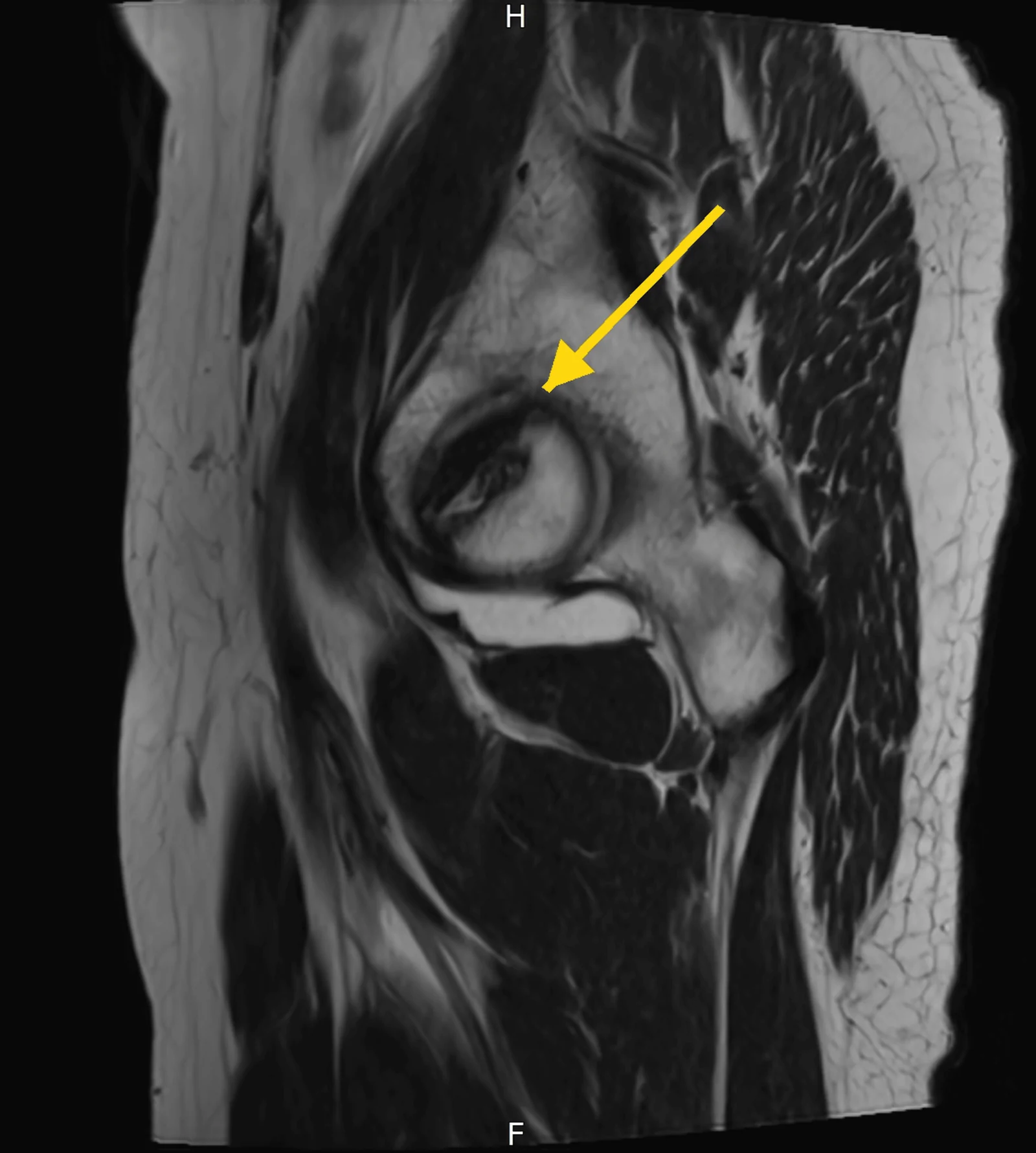
Sagittal MR image of the right hip The arrow indicates the femoral head lesion without subchondral collapse, consistent with Ficat-Arlet stage II disease.

Therefore, MRI of the hips was performed (Figures 2, 3). MRI revealed osteonecrosis of the right femoral head with bone marrow edema and mild osteoarthritic change of the right hip joint with chondromalacia and a large joint effusion and synovitis. Remarkably, no musculotendinous abnormalities were found, suggesting that the persistent soft tissue injury could not explain the symptoms. The left hip joint demonstrated mild chondromalacia with a small joint effusion, but no labral tears were identified in both hips. Based on the radiographic and MRI findings, the lesion was staged as Ficat-Arlet stage II with preserved femoral head contour and no subchondral collapse and crescent sign [6,7].

Due to the persistence of pain and MRI findings, the patient was referred to an orthopedic surgeon who recommended operative treatment. The specific procedure and its further results were performed at the referral facility and are unknown to us.

## Discussion

This case is important for two reasons: the seemingly trivial initial trauma and the seven-month diagnostic delay based on the normal initial radiographs.

First, traumatic osteonecrosis of the femoral head usually occurs after a high-energy trauma, such as a femoral neck fracture or hip dislocation, with a disruption of the vessels supplying the femoral head [4,5]. For example, Rubinstein and Beals described 31 young adults who developed osteonecrosis following a femoral neck fracture and experienced considerable long-term morbidity [5]. In our patient, there was only a minimal twisting trauma, without any fractures or dislocations. There are two possible explanations: first, the trauma could have caused a microvascular injury and contributed to the development of the disease, or second, osteonecrosis may have been idiopathic, and the injury could have just unmasked the symptoms of a previously damaged hip [1,13]. The present case does not help differentiate between these two possibilities, and we do not try to suggest that there is any causality here. Epidemiological data at the national level show that a portion of osteonecrosis cases is not associated with either a history of systemic corticosteroid exposure or chronic alcohol abuse [14].

Second, the diagnostic delay in this case was based on a normal radiograph taken shortly after symptom onset. It is a widely known diagnostic pitfall: osteonecrosis is typically radiographically occult in early stages, and Coleman et al. demonstrated that MRI helps detect avascular necrosis in hips with negative radiographs [11]. Mitchell et al. further showed that MRI findings correlate with the radiographic staging and clinical status, confirming its usefulness as a diagnostic modality [10]. In our patient, the course of radiography is informative by itself: a radiograph obtained at the initial presentation was normal, while the radiograph at presentation seven months later demonstrated degenerative change and features suspicious for osteonecrosis, as expected in a natural radiographic course of the disease and its insensitivity to plain films at early stages [6,10,15]. In our patient, MRI not only confirmed the diagnosis, but also did not reveal any musculotendinous abnormalities, suggesting that a soft-tissue injury was unlikely to cause the pain. A minor injury to a muscle or tendon at the moment of injury is also unlikely to be excluded retrospectively. The bone marrow edema, found at the same time, is also a known correlate of pain in the early stages of the disease [16]. The important thing is that the initial management of the patient at the outside clinic was correct given the normal radiograph and the minimal-energy trauma. The delay in the case is based on the normal radiograph, which was reassuring and resulted in the patient's decision not to continue his medical treatment. The lesson here is that the persistence of hip pain and normal radiographs should be considered as an indication for re-evaluation and performing an MRI scan. Early diagnosis is important since osteonecrosis, if left untreated, usually results in femoral head collapse [15,17], and joint-preserving procedures, such as core decompression, are more effective in the pre-collapse (Ficat-Arlet stage I-II) disease [2,4,18]. In this respect, the mild osteoarthritic changes in the affected hip at diagnosis may represent early secondary joint deterioration, but degenerative changes to this degree cannot be definitely linked to the period of diagnostic delay in a single case.

The presence of mild cartilage changes in the unaffected hip is of uncertain significance. It is known that unilateral hip disease may affect the load on the opposite hip [19,20], but asymptomatic cartilage changes are quite common among young adults, and no conclusion can be made about a single patient; we report the finding without an interpretation. Without fracture or dislocation, direct vascular disruption is unlikely, and a subclinical lesion becoming symptomatic during running is at least as plausible. The initial radiographs from the outside facility were not available for independent review, and their normal interpretation rests on the referral records and the patient's account; subtle sclerosis or lucency may have been present but not appreciated. As unilateral osteonecrosis may later involve the opposite hip, clinical follow-up with a low threshold for MRI of the contralateral hip is advisable.

## Conclusions

Osteonecrosis of the femoral head can develop in a previously healthy young adult in the setting of a minimal twisting injury, and the early disease can be radiographically occult. As a single case report, the temporal association does not establish causality. Persistence of hip pain that does not improve with conservative treatment should increase suspicion of osteonecrosis and prompt the performance of MRI regardless of normal initial radiographs and the absence of classic risk factors. Early diagnosis ensures the widest choice of joint-preserving treatment options. Clinical and MRI surveillance of the contralateral hip is also recommended, given the potential for bilateral involvement.
